# The eptinezumab:CGRP complex structure – the role of conformational changes in binding stabilization

**DOI:** 10.1080/21655979.2021.2006977

**Published:** 2021-12-11

**Authors:** Laurent David, Michelle Scalley-Kim, Andrea Olland, Andre White, Kira Misura

**Affiliations:** a Computational Chemistry and Structural Biology, H. Lundbeck A/S, Valby, Copenhagen, Denmark; b Protein Engineering, Seagen, Bothell, WA, USA; c Crystallography (Andrea Olland) and C.S.O. (Andre White), Xtal BioStructures, Inc, Natick, MA, USA; d Research & Development, Lundbeck Seattle BioPharmaceuticals, Inc, Bothell, WA, USA

**Keywords:** Eptinezumab, CGRP, calcitonin gene-related peptide, migraine, anti-CGRP, monoclonal antibody

## Abstract

To further elucidate the mechanism of action and binding properties of eptinezumab to calcitonin gene-related peptide (CGRP), X-ray crystallography, computational alanine scanning, and molecular dynamics were used. X-ray diffraction data were collected to determine the three-dimensional structures of the unbound eptinezumab antigen-binding fragment (Fab) and the Fab:CGRP complex. Molecular dynamics simulations were performed to analyze the transition between uncomplexed and complex states. The amidated C-terminus of CGRP was shown to bind in a pocket formed by the Fab heavy and light chains. There was extensive contact between all six complementarity-determining regions (CDRs; composed of light-chain [L1, L2, and L3] and heavy-chain [H1, H2, H3]) of eptinezumab and CGRP. The complex demonstrated a high ligand-binding surface area dominated by aromatic residues. CDR L3 contains a disulfide bond that stabilizes the loop, contributes surface area to the binding pocket, and provides van der Waals contacts. Comparison of the uncomplexed and complex structures revealed motion near the binding cleft. The CDR loops H2 and H3 were displaced ~1.4–2.0 Å and residue H-Tyr33 changed conformation, creating a ‘latch-and-lock’ mechanism for binding CGRP and preventing dissociation. Computational alanine scanning of CGRP identified energetic ‘hot spots’ that contribute to binding energy; mutating these positions to residues in homologous neuropeptides resulted in unfavorable binding energies. The attributes of the Fab region and the conformational changes that occur in eptinezumab during binding to CGRP contribute to the specificity, durability, and strength of the interaction, and likely underlie the rapid and sustained migraine preventive effect observed in clinical studies.

## Graphical abstract

**Figure:**
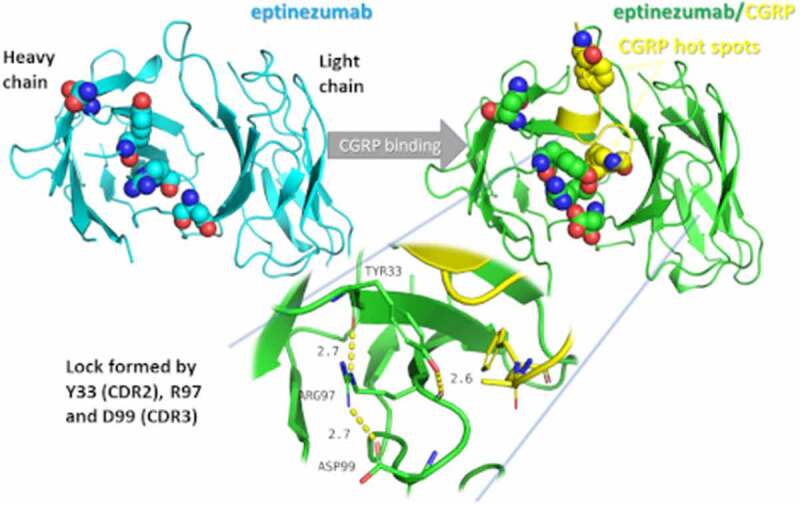

## Introduction

Migraine is a highly prevalent neurologic disorder, affecting an estimated one billion individuals worldwide [1]. The symptoms of migraine can be disabling. They commonly interfere with family, education, and work obligations, and may contribute to the development of comorbidities such as cardiovascular disease, depression, and anxiety [2–9]. In 2016, it was estimated that the global burden of migraine exceeded 45 million patient-years lived with disability, making it the second leading cause of disability worldwide [1].

Calcitonin gene-related peptide (CGRP) plays an important role in migraine pathophysiology [10,11]: Within the trigeminovascular complex, release of CGRP facilitates vasodilation and neurogenic inflammation. CGRP promotes enhanced neuronal activity both within the trigeminal ganglion and at the neurovascular interface, which can lead to central sensitization [12,13].

The humanized immunoglobulin G1 monoclonal antibody eptinezumab is an antagonist of the CGRP ligand [14] indicated for the preventive treatment of migraine in adults [15]. It was intentionally developed for intravenous administration; the marketed formulation provides 100% bioavailability and rapid attainment of maximal plasma concentrations. The migraine preventive effects of eptinezumab have been demonstrated across the migraine spectrum, with statistically significant reduction from baseline in migraine frequency beginning as early as one day after the initial infusion and continuing throughout the 12-week dosing interval [16–20].

Eptinezumab binds CGRP with high affinity and selectivity [14,21], and provides sustained blockade of the ligand’s interaction with its receptor and associated vasodilation and dysfunctional activation within the trigeminovascular system [10,22]. Thus, we predict that there are structural components and specific residues that underlie this affinity and selectivity. Here we sought to determine the structure of eptinezumab in its complexed and uncomplexed state with CGRP and significant residues involved in the complex, as well as to understand the molecular details of the interaction between CGRP and eptinezumab and traits of a high affinity monoclonal antibody to aid in future discovery efforts in this space. The objective of this work was to further elucidate the mechanism of action and binding properties of eptinezumab to CGRP using X-ray crystallography, computational alanine scanning, and molecular dynamics simulations.

## Methods

### Fab generation

The antigen-binding fragment material was produced by partial endoproteolysis of eptinezumab with immobilized papain. Fragments were purified by Fc depletion followed by size-exclusion chromatography (SEC). Purity was assessed by SEC and sodium dodecyl sulfate-polyacrylamide gel electrophoresis (SDS-PAGE).

### Fab:antigen complex and uncomplexed Fab purification and crystallization

α-CGRP (human) 37-mer peptide when mature (Bachem; amino acid numbering used in this paper is for the pro-hormone sequence of the peptide except where specified) (hereinafter CGRP) was mixed with the Fab fragment. The Fab:antigen complex was purified by SEC, concentrated, and screened for crystallization. Conditions that initially produced microcrystals were identified and optimized to improve the crystal size and quality. Crystals grew after one week at 20°C in sitting drops equilibrated against a solution of 26% PEG MME (polyethylene glycol monomethyl ether) 550, 9.5 mM ZnSO_4_, 95 mM MES (2-(N-morpholino)ethanesulfonic acid) pH 6.5, and 30 mM glycyl-glycyl-glycine.

Originally, the uncomplexed Fab failed to crystallize with broad sparse matrix screening. Random matrix microseeding was undertaken with a seed stock generated from Fab:CGRP complex crystals. Here, uncomplexed Fab crystals were obtained by sparse matrix screening with seeding (using the Fab seed stock generated previously). Fab crystals grew in sitting drops containing 300 nL Fab at 15 mg/mL, 200 nL reservoir solution, and 100 nL of seed stock. The reservoir solution contained 23% PEG MME 2000, 100 mM Tris pH 8.5, and 200 mM MgCl_2_. Crystals grew after one week of incubation at 20°C.

Both Fab:CGRP complex crystals and Fab crystals were harvested in microfiber loops and drawn through a cryoprotectant solution containing 20% glycerol (Fab:CGRP crystals) or 25% glycerol (Fab crystals) in reservoir solution before being flash-cooled in liquid nitrogen.

### Structure determination

Crystal structures were determined using standard X-ray diffraction techniques. X-ray diffraction data for Fab:CGRP complex were measured at the Australian Light Source in Clayton, Australia, using a Dectris Eiger X 16M detector. X-ray diffraction data for the uncomplexed Fab were measured at beamline BL13-XALOC at the ALBA synchrotron in Cerdanyola del Vallès, Barcelona, Spain, and data were collected using a Dectris Pilatus 6M. For Fab:CGRP data, processing was performed using X-ray Detector Software (XDS) [23] for indexing and data reduction, then merged, scaled, and averaged with AIMLESS from the CCP4 suite of programs (hereinafter referred to as CCP4). For the uncomplexed Fab data, processing was performed using HKL2000 [24], then merged, scaled, and averaged with SCALEPACK (component of HKL2000). Diffraction statistics are summarized in Table 1.

**Table 1.** X-ray diffraction statistics

	Fab:CGRP	Fab
Wavelength (Å)	0.95372	0.97917
Space group	*C*2	*P*2_1_22_1_
Unit cell parameters (*a, b, c* in Å; α, β, γ in °)	*a* = 68.235, *b* = 207.104, *c* = 68.204; α = γ = 90.00, β = 91.08	*a* = 62.781, *b* = 83.485, *c* = 90.190; α = β = γ = 90.00
Resolution (Å)	48.53–3.10 (3.31–3.10)	50.00–1.54 (1.60–1.54)*
Measured reflections	66,115 (11,076)	423,952
Unique reflections	17,052 (2,996)	70,440 (6,946)
*R*_sym_	17.4 (64.6)	0.054 (0.823)
Completeness (%)	99.2 (96.7)	99.8 (99.9)
Average I/σ	6.0 (1.7)	21.7 (1.4)
Redundancy	3.9 (3.7)	6.0 (5.7)
CC_1/2_	0.984 (0.665)	0.999 (0.639)
Fab molecules per asymmetric unit	2	1

*Highest resolution shell of 3.31–3.10 Å (Fab:CGRP) and 1.60–1.54 Å (Fab).

Abbreviations: CC_1/2,_ correlation coefficient determined by two random half-sets of data; CGRP, calcitonin gene-related peptide; Fab, antigen-binding fragment; *R*sym, agreement between symmetry equivalent reflections.

**Table 2.** Model refinement statistics

	Fab:CGRP	Fab
Refinement resolution (Å)	48.57–3.10	45.14–1.54
*R*_cryst_ (%)	18.05	15.9
*R*_free_ (%)	26.58	19.0
Protomer details (per asymmetric unit) Eptinezumab Fab CGRP	2 2	1 ‒
Bond lengths, rms (Å)	0.005	0.016
Bond angles, rms (°)	1.467	1.99
Ramachandran plot (%) Preferred Allowed Outliers	86.9 9.9 3.2	97.4 2.6 0.0

Abbreviations: CGRP, calcitonin gene-related peptide; Fab, antigen-binding fragment

**Table 3.** Main epitope/paratope interactions

Eptinezumab	Atom	Chain	CGRP	Atom	
**# H-bond**					
Ser92	OG	L3	Ser116	O	P
Tyr37	OH	L	Phe119	O	P
IIe53	N	H2	Asn113	O	P
Asn54	N	H2	Asn113	OD1	P
Gln47	NE2	L	Ala118	O	P
**# Water-mediated H-bond (putative)**					
Ser92	OG	L3	Phe119Cter	N	P
Tyr93	O	L3	Ser116	OG	P
Cys100	O	L3	Ser116	OG	P
Asp51	O	L2	Lys117	O	P
**# Main hydrophobic contacts**					
Cys95	SG	L3	Val114	CG2	P
Cys95	SG	L3	Phe109	CE1	P
Tyr58	CD2	H	Val114	CG1	P
Val50	CG1	H	Val114	CB	P
Val37	CG2	H	Phe119	CZ	P
Leu90	CD2	L3	Phe119	CG	P
Tyr33	CE1	L1	Lys117	CD	P
Tyr29	CE1	L1	Pro111	CG	P
Tyr33	CE2	L1	Pro111	CB	P
**# Main pi-pi interactions**					
Asn54		H2	Asn113		P
Asn35		H	Phe119		P
Phe101		L2	Phe119		P
Tyr33		H1	Val114		P
Arg97		H3	Phe119		P
Tyr29		L1	Phe109		P
Asp94		L3	Phe109		P

Abbreviations: CGRP, calcitonin gene-related peptide; Cter, C-terminus.

For the Fab:CGRP structure, two initial models of a Fab, not including complementarity-determining regions (CDRs), were placed using molecular replacement (MR) with Phaser [25] (CCP4). The final model including CDR regions and CGRP was built with iterative cycles of model building in Coot [26] and refinement in REFMAC5 [27] (CCP4). The Fab portion of this structure with CDRs and antigen removed was used in MR to obtain an initial model for the Fab alone. The final model was built as above, with Coot and REFMAC5. Final refinement statistics are shown in Table 2. Fab ligand-binding surface area across heavy and light chains was calculated using QtPISA (CCP4). Both the solvent-accessible surface area and the interface surface area of the Fab:antigen interaction were calculated.

The Rosetta software suite was used to calculate the binding energies for the wild-type and mutant complexes as previously described [28]. Briefly, in comparison to other methods, this method integrates movement in both the neighboring side chains and backbone torsion angles for positions within 8 Å of the mutation. This computational flexibility more closely mimics adjustments that would likely occur because of mutation, and in a test set of 1240 mutations across a variety of protein-protein interfaces the method was shown to have a Pearson correlation factor (R) of 0.63 and to correctly predict 76% of stabilizing and destabilizing mutations. To conserve computing power, 10 iterations of the structure optimization, minimization, and scoring process were completed for the CGRP alanine mutations and 35 iterations for the multiple mutations, as opposed to 50 indicated by Barlow *et al.* [28].

Molecular dynamics simulations were performed to analyze structural differences between uncomplexed and complex states. Maestro (Schrödinger Release 2020–4: Maestro, Schrödinger, LLC, New York, NY, 2021) was used to 1) prepare the structure using the ‘Protein Preparation’ panel; 2) add the water molecules using Maestro panel ‘System Builder’ with default parameters (i.e., solvent model SPC, orthorhombic box as boundary conditions with a buffer of 10 Å; and 3) set the simulation parameters using the ‘Molecular Dynamics’ panel setting the simulation time to 250 ns and using default parameters (i.e., using the ensemble class NPT for the production run at 300 K with a pressure of 1.01325 bar, the Nose-Hoover chain thermostat method with a relaxation time of 1 ps and the Martyna-Tobias-Klein barostat method with a relaxation time of 2 ps). To increase sampling diversity, four simulations of 250 ns were performed for each state using different seeds. Because the uncomplexed structure did not have CDR H1 resolved, and both structure states were very similar, the CGRP peptide was omitted from the bound structure which in turn was used as the initial structure for the uncomplexed form. Watermap [29,30] (Schrödinger Release 2020–4: Maestro) was used to analyze where water molecules could sit in the Fab:CGRP complex using default parameters.

## Results

**Figure 1.**
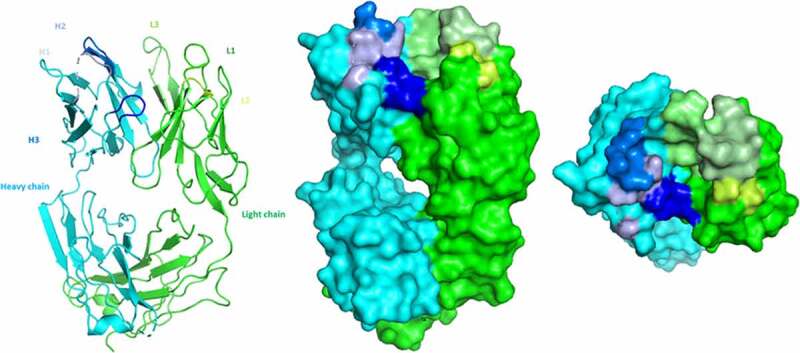
Crystal structure of eptinezumab in its free (unbound) state, in which the heavy chain (cyan) and the light chain (green) are shown from the side and top views. The CDRs are colored using PyMOL color names, where light chain regions are in shades of green (L1 [pale green], L2 [lemon], L3 [lime]) and heavy chain regions are in shades of blue (H1 [light blue], H2 [marine], H3 [blue])

**Figure 2.**
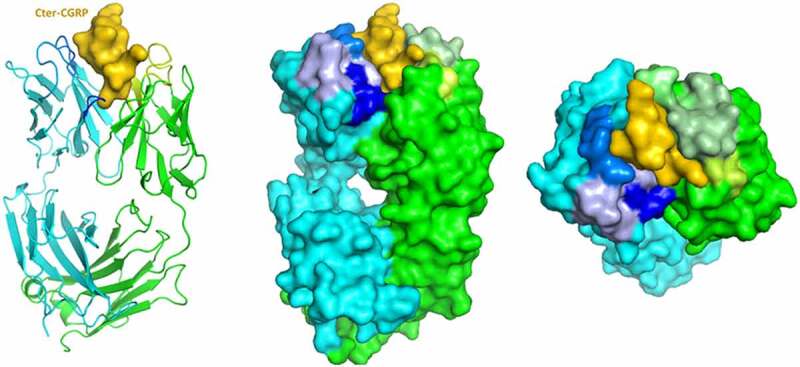
Crystal structure of the eptinezumab:CGRP complex. CGRP (yellow) is shown bound in the deep pocket formed by the light (green) and heavy (cyan) chains of the Fab. The CDRs are colored using PyMOL color names, where light chain regions are in shades of green (L1 [pale green], L2 [lemon], L3 [lime]) and heavy chain regions are in shades of blue (H1 [light blue], H2 [marine], H3 [blue])

We predict that there are structural components and specific residues that contribute to the affinity and selectivity of eptinezumab binding to CGRP. Our aims were to determine the structure of eptinezumab in its uncomplexed state and complexed with CGRP, and to elucidate the mechanism of action and binding properties of eptinezumab to CGRP. We used X-ray crystallography, computational alanine scanning, and molecular dynamics simulations. The structures of eptinezumab in its uncomplexed state and as a complex with CGRP are presented in Figures 1–2, respectively. In the complexed eptinezumab:CGRP, CGRP amino acids 26–37 (mature hormone numbering; corresponds to 108‒119 of pro-hormone) only are visible in the structure; residues 1–25 are presumed disordered and not included in the structure. Analysis of the atomic resolution of the eptinezumab:CGRP complex structure reveals that the amidated C-terminus of CGRP binds in a deep, narrow pocket formed by the eptinezumab Fab heavy and light chains. The complex structure has a ligand binding surface area of 797 Å^2^ total (320 Å^2^ and 477 Å^2^ contributed from heavy and light chains, respectively; calculated using QtPISA) which is dominated by aromatic residues; these aromatic side chains likely drive the selectivity and specificity of the paratope. All six complementarity-determining regions (CDRs) of eptinezumab (H1, H2, H3, L1, L2, L3) make extensive contacts with CGRP; this paratope area consists of multiple secondary structure elements, including a helical turn and a network of intra hydrogen bonds.

**Figure 3.**
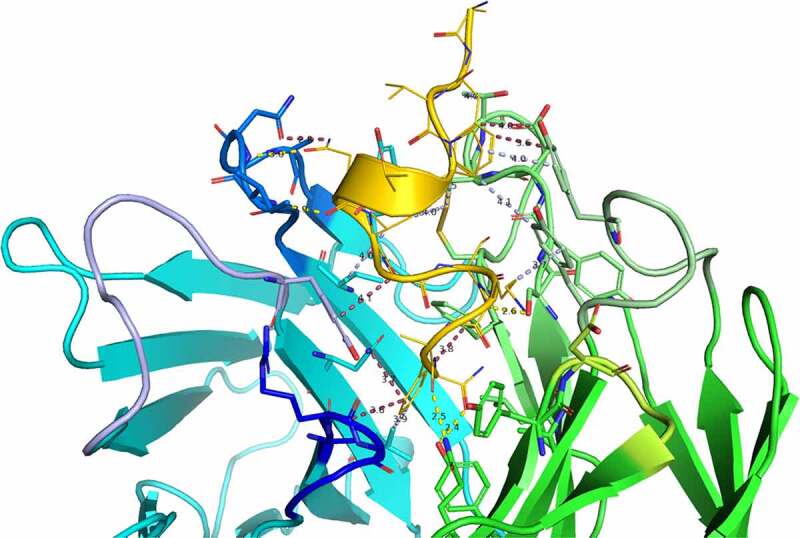
Detailed interactions between CGRP and eptinezumab. Hydrogen bond in yellow, hydrophobic contact in pale green, and pi-pi interactions in raspberry. The CDRs are colored such that light chain regions are in shades of green (L1 [pale green], L2 [lemon], L3 [lime]) and heavy chain regions are in shades of blue (H1 [light blue], H2 [marine], H3 [blue]). All colors are PyMOL color names

Detailed interactions between CGRP and eptinezumab are illustrated in Figure 3. The epitope/paratope interactions consist of five hydrogen bonds and numerous hydrophobic interactions (van der Waals contacts), and seven important pi-pi interactions, most of which are between CGRP and the CDRs (Table 3, Supplementay Figure 1). Notably, all five CGRP-eptinezumab hydrogen bonds are donated by the paratope to the epitope according to the crystal structure. CGRP’s amidated Phe119 C-terminal amide accepts a hydrogen bond from L-Tyr37 hydroxyl and donates a hydrogen bond internally to Ser116 main chain carbonyl oxygen. The latter internal interaction is thought to induce a helical turn in the CGRP C-terminal region such that it fits well in the binding site. This secondary structural element is further stabilized by Ala118 and Ser116 carbonyl oxygens accepting a hydrogen bond from L-Gln47 side chain amide and L-Ser92 hydroxyl side chain, respectively. The crystal structure further clarifies the importance of CGRP’s Asn113, in which its side chain amide carboxyl oxygen accepts a hydrogen bond from CDR H2 Asn54 main chain nitrogen and its carbonyl oxygen accepts a hydrogen bond from H-Ile53 main chain nitrogen (Supplementary Figure 2).

**Figure 4.**
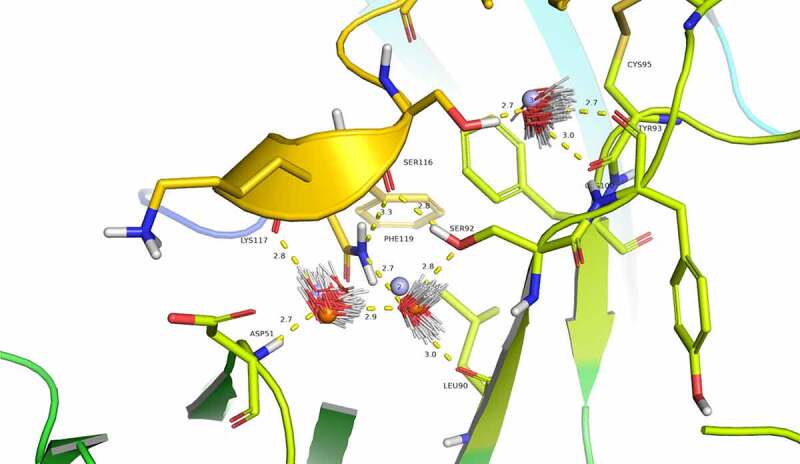
Evidence for structural water molecule in eptinezumab:CGRP interface. CGRP is colored in yellow and CDR L2 in Limon, using PyMOL color names. The locations of water molecules from the X-ray crystallography structure of the uncomplexed eptinezumab (orange spheres), of water molecules obtained by Watermap from the complex structure (light blue spheres 1–3), and of explicit clusters of water molecules obtained by Watermap from the eptinezumab:CGRP complex (HOH clusters). Watermap places 2 water molecules (light blue spheres 1 and 2) in a cavity within the bound structure, forming a network of hydrogen bond between Asp51 NH (CDR L2) to water1, water1 to water2, water2 to Leu90 C = O and Ser92 OG (CDR L3), and finally Ser92 OGH to Ser116 C = O (CGRP); CGRP Ser116 OGH does not make any direct interaction with eptinezumab but faces another small cavity. Watermap placed one water molecule (light blue sphere 3) in this cavity, indicating a hydrogen bond network with O from water3 and water3 to Cys100 and Tyr93 (CDR L3) carbonyls

Although not clearly identifiable in the experimental structure due to resolution limitations, analysis using Watermap predicts three water molecules linking CGRP to eptinezumab light chain through a network of hydrogen bonds (Figure 4). Water1 and water2 overlap well with two water molecules from the unbound eptinezumab X-ray structures (shown in orange in Figure 4). Watermap calculations performed on the unbound structure (bound eptinezumab structure with removed CGRP) predict the same hydration sites as those identified in the complex (spheres in light blue, Figure 4). This observed overlap indicates that hydration sites for the uncomplexed eptinezumab are conserved in the eptinezumab:CGRP complex and suggests that water molecules are mediating interactions between CGRP and eptinezumab. The CGRP terminal amide nitrogen from amidated-Phe119 donates a weak hydrogen bond to CGRP Ser116 carbonyl oxygen (distance = 3.3 Å) and faces a cavity. In this cavity, Watermap places two water molecules (light blue spheres 1 and 2; Figure 4) where a network of hydrogen bonds forms between Asp51 main chain nitrogen (CDR L2) and water1, water1 to water2, water2 to Leu90 carbonyl oxygen and Ser92 hydroxyl (CDR L3), and Ser92 hydroxyl to Ser116 carbonyl oxygen (CGRP). CGRP Ser116 hydroxyl does not form any direct interaction with eptinezumab but faces another small cavity where Watermap predicts the placement of one water molecule (light blue sphere 3, Figure 4), indicating there may be a hydrogen bond network with Ser116 from water3 and from water3 to Cys100 and Tyr93 (CDR L3) carbonyls.

**Figure 5.**
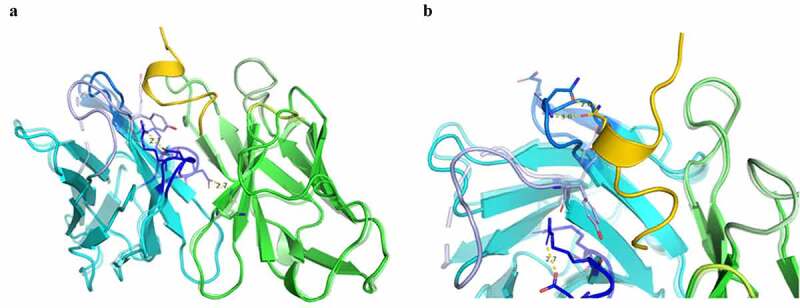
Comparison of bound and unbound structures indicate conformational changes during eptinezumab binding to CGRP. Eptinezumab is in yellow. The CDRs are colored using PyMOL color names, where light-chain regions are in shades of green (L1 [pale green], L2 [lemon], L3 [lime]) and heavy-chain regions are in shades of blue (H1 [light blue], H2 [marine], H3 [blue]). Superposed structure using the FV domain as template a) of the unbound (translucent) and bound (opaque) structure. CDR H3 (blue) clearly goes through a structural reorganization where an internal H-bond between Asp99 and Arg97 in the bound form goes to an H-bond between Asp99 and light chain Lys46 inducing a movement of Asp99 CA by ~3 Å (from 2.5 Å to 3.5 Å following the alignment); b) of the unbound (translucent) and bound (opaque). Asn54 rotates and makes a direct contact with CGRP Asn113. Although the angles between both amides is not right for a proper hydrogen bond, the inter distance between N-Asn113 and O from Asn54 CDR H2 is 2.8 Å

Comparison of the Fab:CGRP complex and uncomplexed Fab structures suggests that conformational changes occur upon eptinezumab binding to CGRP (Figure 5). Although the structures are similar overall, a small rigid body movement occurs. The largest changes were observed near the CGRP binding cleft, such as the displacement of the heavy-chain CDR loops H2 and H3 by about 1.4–2.0 Å and conformation changes in residue H-Tyr33. These changes result in the formation of a hydrogen bond network, notably involving Arg97 and Asp99.

These conformational changes create a ‘latch-and-lock’ mechanism for binding the CGRP ligand, thus hindering dissociation (**Movie**). Molecular dynamics simulations indicate conformational freedom in Tyr33 (Supplementary Figure 3), supporting the hypothesis that this residue can allow entry of CGRP into the binding cleft where CGRP C-terminus Phe119 points straight down into and fits with hydrophobicity complementarity (Supplementary Figure 4a-d). Specifically, molecular dynamics simulations of the uncomplexed Fab (4,000 frames) yielded a conformation of Tyr33 resembling the uncomplexed and complexed Fab structure in ~50% and ~25% of the molecular dynamics frames, respectively. In the remaining ~25% of frames, the conformation of Tyr33 was observed to be between the uncomplexed and complex conformations, but still overlapping CGRP (Supplementary Figure 3b). The simulations results with the uncomplexed Fab also showed that Tyr33 oscillated between these three positions (chi1 = ‒60°, 60°, and 180°; Supplementary Figure 3a), while the simulations results with the complexed Fab have more than 99% of the frames where Tyr33 adopts the initial formation (–54°), further suggesting that this residue Tyr33 is a key component of the lock process.

**Figure 6.**
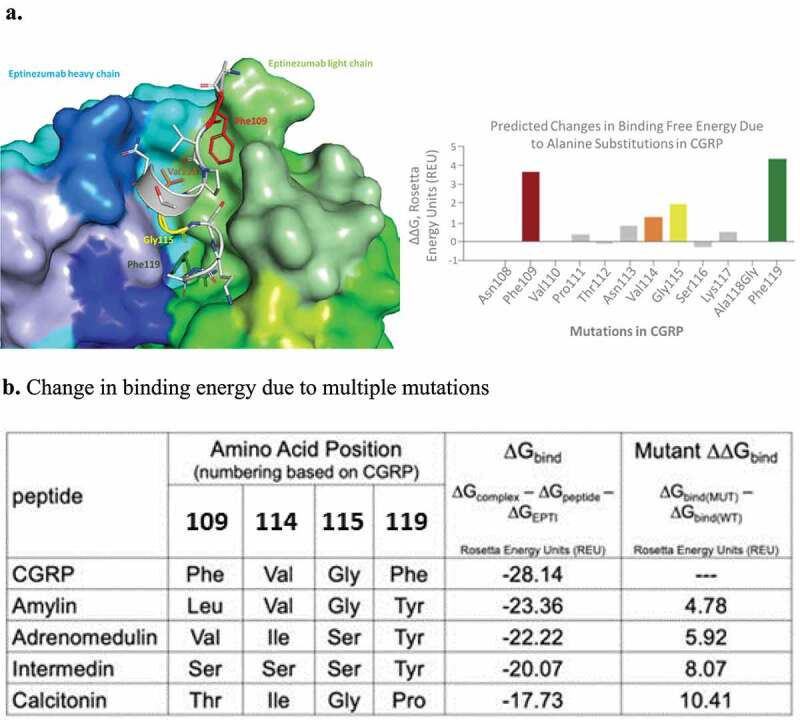
Computational alanine scanning of CGRP identifies key interface residues. a) The CDRs are colored using PyMOL color names, where light-chain regions are in shades of green (L1 [pale green], L2 [lemon], L3 [lime]) and heavy-chain regions are in shades of blue (H1 [light blue], H2 [marine], H3 [blue]). CGRP is in gray. Four positions in CGRP are important for binding: Phe109 (red), Val114 (Orange), Gly115 (yellow), Phe119 (green). b) Table showing loss in binding energetics observed when these key positions mutated to the corresponding amino acid in related neuropeptides based on sequence alignment

In order to better understand the basis of eptinezumab specificity and selectivity for CGRP, we used computational alanine scanning to identify critical ‘hot spot’ amino acids in CGRP that contribute to the binding energy (Figure 6a). Analyses of the results suggest that GCRP’s epitope is its C-terminal region where Phe109 and Phe119 are key contributors. These two Phe residues are unique to CGRP when compared to other related neuropeptides, thus suggesting a rationale for the selectivity of eptinezumab versus related peptides. Unsurprisingly, mutating these key positions in CGRP to residues found in homologous peptides (i.e., adrenomedullin, intermedin, calcitonin, and amylin) resulted in unfavorable binding energies (Figure 6; Supplementary Figure 5).

## Discussion

The Fab region of eptinezumab has some unique attributes that likely contribute to the specificity, durability, and strength of binding to CGRP, including a small CDR H3, a unique disulfide bond in CDR L3, and a deep narrow hydrophobic cleft. The small CDR H3 helps create space between the heavy- and light-chain interface, enabling a deep pocket for CGRP binding. The disulfide bond in CDR L3 (a feature more prevalent in antibodies derived from rabbit sources), which stabilizes this loop, likely contributes surface area to the binding pocket and provides van der Waals contacts for the CGRP ligand. The hydrophobic cleft is where the CGRP C-terminus fits straight down into, with good shape and hydrophobicity complementarity, and contributes to binding selectivity [31]. These attributes, and the interaction and contact of CGRP to all six of eptinezumab’s CDRs, support previous reports on the high selectivity and affinity [14] of eptinezumab for CGRP.

Comparison of the uncomplexed eptinezumab and eptinezumab:CGRP complex revealed key differences near the binding cleft. The heavy-chain CDR loops H2 and H3 were displaced by about 1.4–2.0 Å; residue H-Tyr33 changed conformation; and a new hydrogen bond network was formed between Tyr33, Arg97, and Asp99. These conformational changes appear to create a latch-and-lock mechanism for binding the CGRP ligand and preventing dissociation. The conformational freedom of Tyr33 likely plays an important role in facilitating the entry of CGRP into the binding cleft.

*In silico* techniques support the high selectivity of eptinezumab for CGRP. Positions in CGRP that were most important for binding to eptinezumab (hot spots identified through in silico alanine scanning) were replaced with corresponding amino acids present in related neuropeptides. Some replacements were clearly too big to fit the binding pocket, resulting in significant atomic clashes (e.g., replacing Phe119 with tyrosine or Gly115 with serine); others resulted in loss of energetically favorable intermolecular forces and, subsequently, binding energy (e.g., Phe109 with valine). The free-energy calculations provide a rationale as to why eptinezumab binds to CGRP but not to other related neuropeptides. These calculations are consistent with experimental data that show negligible or dramatically reduced binding [32].

Furthermore, examining CGRP binding to eptinezumab compared to its receptor complex is useful in understanding the mechanism of action. The full-length CGRP receptor complex has been described using cryo-electron microscopy [33]. The region of CGRP bound to eptinezumab Fab (hormone CGRP amino acids 26–37 [108‒119 in pro-hormone]) forms significant interactions with the CLR-RAMP1-Gs receptor complex (Supplementary Figure 6) [33]. This indicates that eptinezumab binds the same region of CGRP that binds to the receptor, thus rendering CGRP incapable of binding to the CGRP receptor.

## Conclusion

Analysis of the eptinezumab:CGRP complex molecular structure reveals that CGRP binds to eptinezumab in a deep, narrow pocket containing positive electrostatic surfaces and hydrophobic surfaces, with extensive contact between all six CDR loops of eptinezumab and CGRP. Conformational changes in the eptinezumab structure during binding to CGRP facilitate a latch-and-lock mechanism of binding that prevents dissociation. These characteristics are consistent with the specificity, durability, and strength of binding previously reported, and likely underlie the effective and sustained migraine preventive effect observed in clinical studies.

## Supplementary Material

Supplemental Material

## Data Availability

The authors declare that the data supporting the findings of this study are available within the article. The authors may be contacted for further data sharing.

## Research highlights

● Extensive contact between all CDR loops of eptinezumab and CGRP

● Movement near the binding cleft underlies ‘latch and lock’ mechanism

● CGRP contains energetic ‘hot spots’ that contribute to binding energy
