# Supplementary material for: The eptinezumab:CGRP complex structure – the role of conformational changes in binding stabilization
Source: Bioengineered. 2021 Dec 11;12(2):11076–86. doi: 10.1080/21655979.2021.2006977 (PMC8810155; doi:10.1080/21655979.2021.2006977)
Supplement: Supplemental Material [file KBIE_A_2006977_SM0885.docx]

**Supplementary Figure 1.** Epitope/paratope interactions: main interactions between aCGRP and eptinezumab (4X250 ns) from the Maestro “Simulation Interaction Diagram” panel.

**
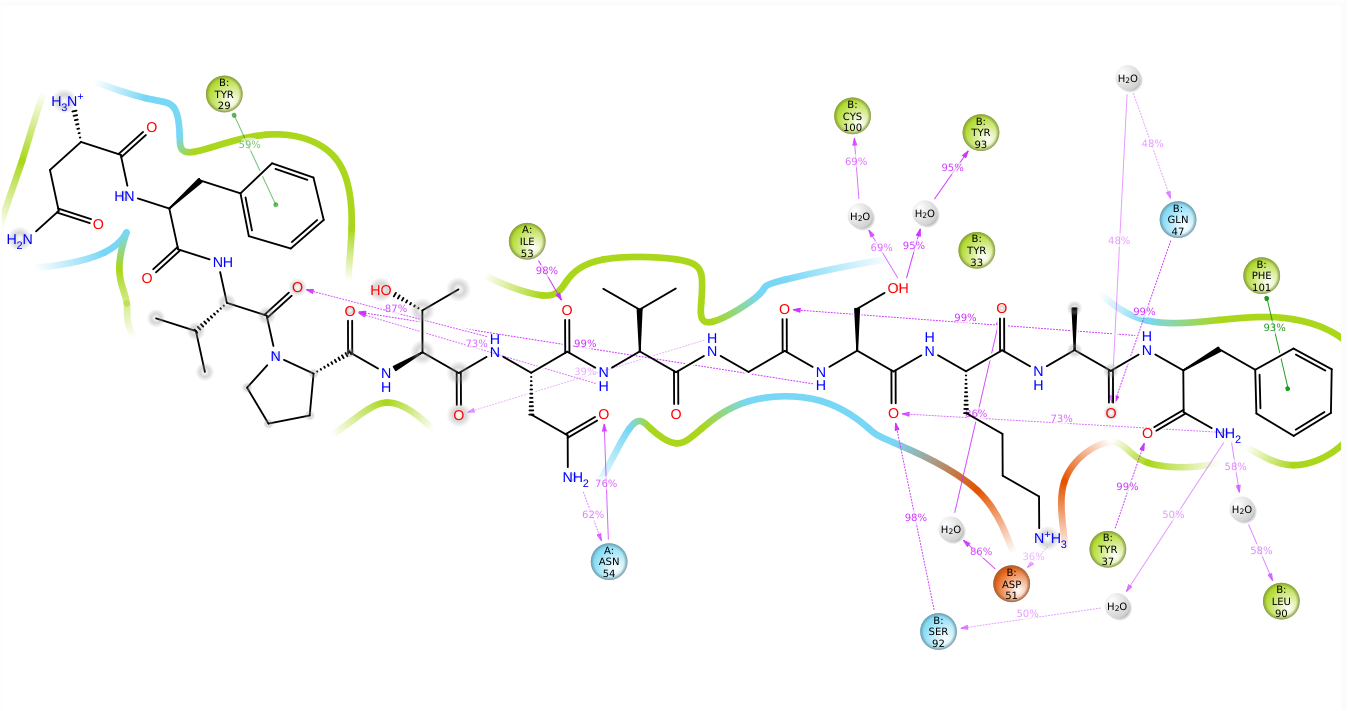
**

**
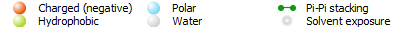
**

**Supplementary Figure 2.** Electron densities (level 1.00) around CDR H2 (blue) showing a hydrogen bond between main chain N Asn54 CDR H2 and CGRP (yellow) Asn113 OD1. Another atypical interaction happens between both amide heads of Asn54 and Asn113.

**
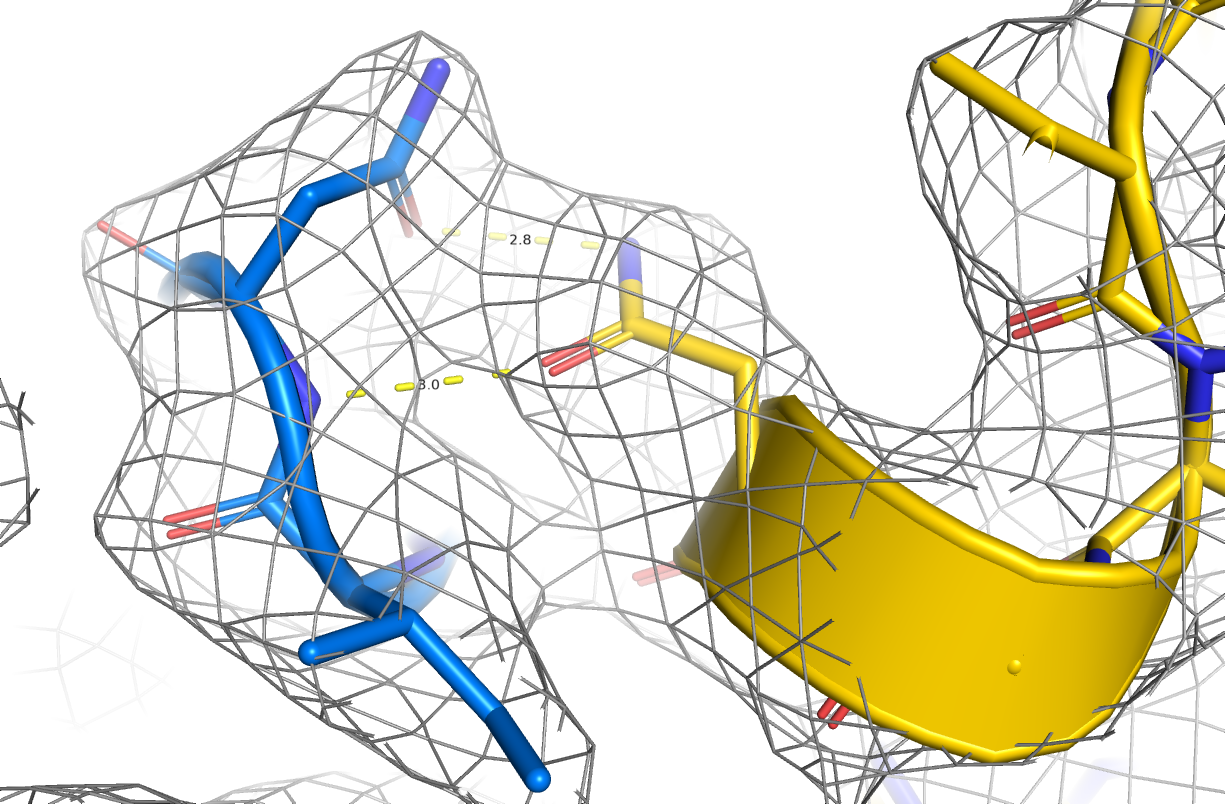
**

**Supplementary Figure 3.** Molecular dynamics simulations (4X250 ns merged) indicative of conformational freedom in Tyr33 (mainly driven by Chi1) for the unbound eptinezumab. **A)** dihedral angle distribution over time over the 4 simulations, 1 dot every 250 ps. **B)** Bar chart distribution, count in y-axis versus binned dihedral angle value in x-axis.

**A B**

**
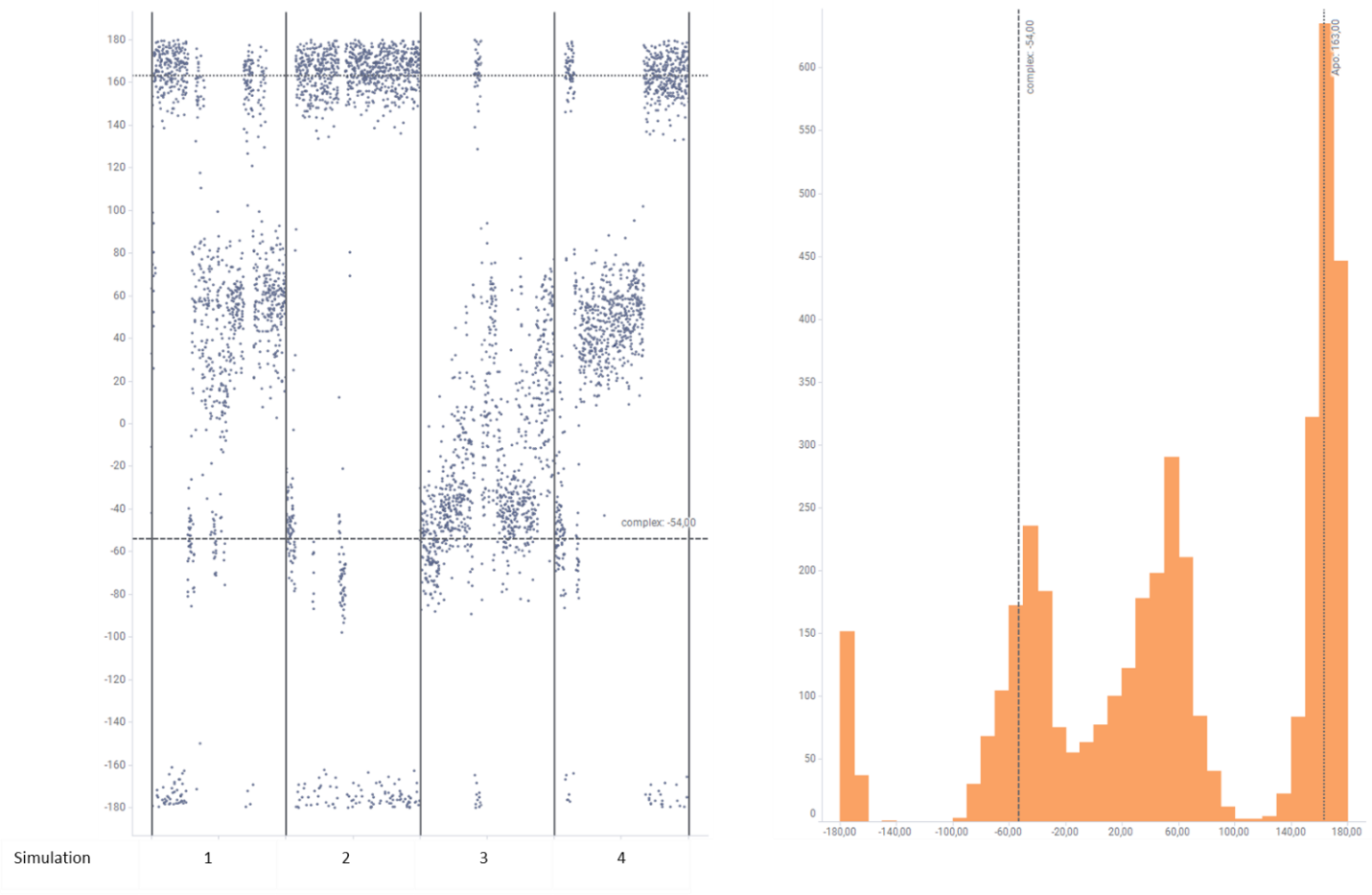
**

**Supplementary Figure 4.** CGRP has shape complementarity and hydrophobic interactions with the deep binding cleft of eptinezumab. **A)** Phe119 binds into a deep and hydrophobic pocket. CGRP in yellow, CDR light chain in green (L1 [pale green], L2 [lemon], L3 [lime]) and heavy chain in cyan (H1 [light blue], H2 [marine], H3 [blue]); **B)** a close-up view of Phe199 complementarity to binding cleft (Fab in cyan and CGRP in yellow); **C)** coloring by Eisenberg hydrophobicity color scale^1^ demonstrates hydrophobic map between Phe119 and the deep cavity; **D)** Tyr33 (Fab uncomplexed in cyan; complex in green) covers CDR H3 in the bound structure and locks Phe119 into the binding pocket. The uncomplexed structure also has a tight cavity covered partly by CDR H3 that needs to open up to accept Phe119.

**A**

**
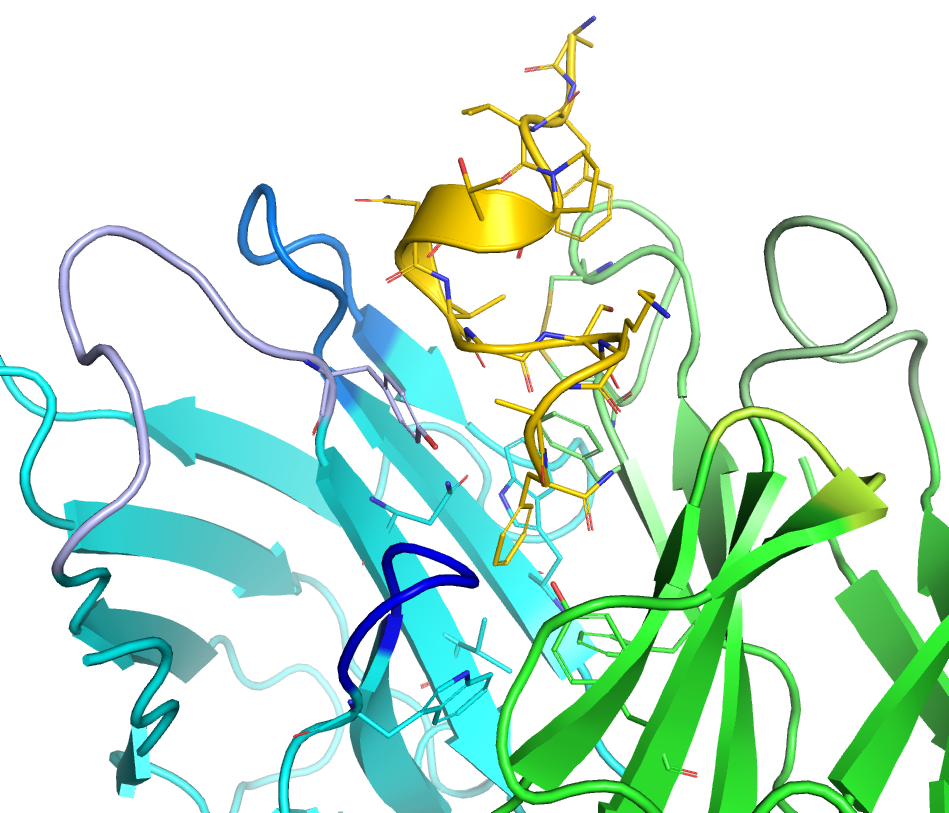
**

**B.**

**
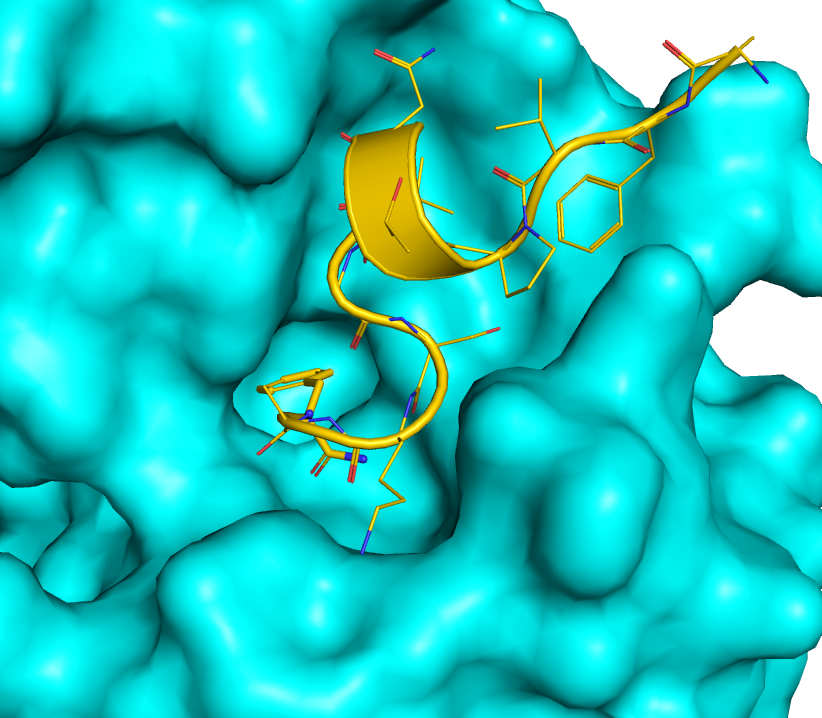
**

**C.**

**
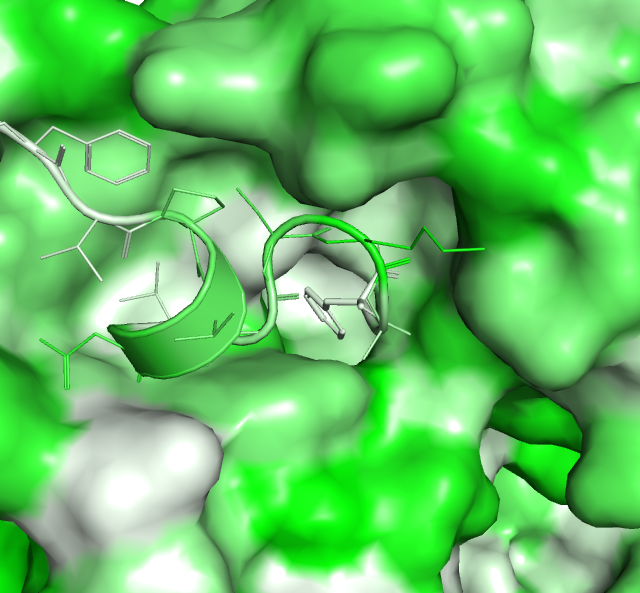
**

**D.**

**
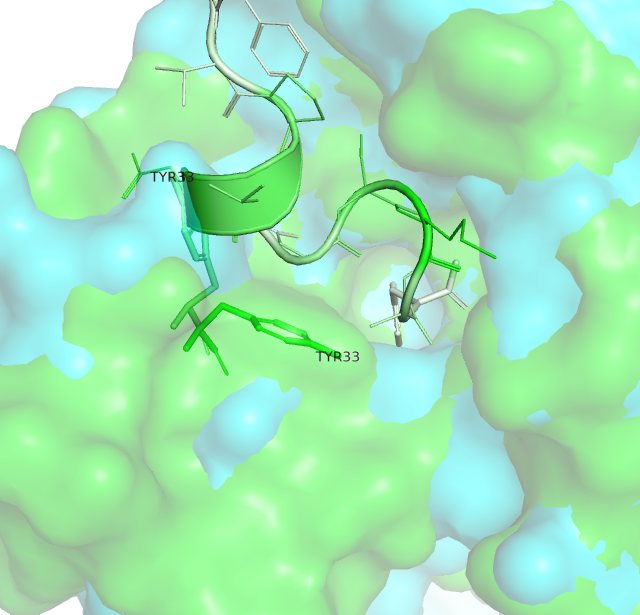
**

**Supplementary Figure 5.** Sequence comparison of CGRP and related peptides for mutations. **A)** Sequence alignment comparing the C-terminal region of CGRP and related neuropeptides with number of amino acids for mature hormone shown. These peptides show 30%‒50% sequence similarity in this region. **B)** Key residues on wild-type CGRP are shown in green (numbering for the pro-hormone listed): Phe109 (F), Val114 (V), Gly115 (G), Phe119 (F) and replaced with corresponding residues from related neuropeptides (in red) to test binding energy to eptinezumab Fab.

**A**

**
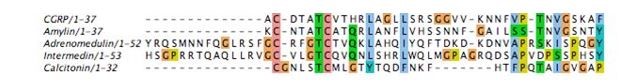
**

**B**

| Wild-type CGRP | NFVPTNVGSKAF |
| --- | --- |
| With amylin residues | NLVPTNVGSKAY |
| With adrenomedulin residues | NVVPTNISSKAY |
| With intermedin residues | NSVPTNSSSKAY |
| With calcitonin residues | NTVPTNIGSKAP |

**Supplementary Figure 6.** Comparison of CGRP C-terminus in bound eptinezumab Fab:CGRP right complex with cryoEM CGRP-CLR-RAMP1-Gs structure^2^ (left) indicates that binding is mutually exclusive.

**
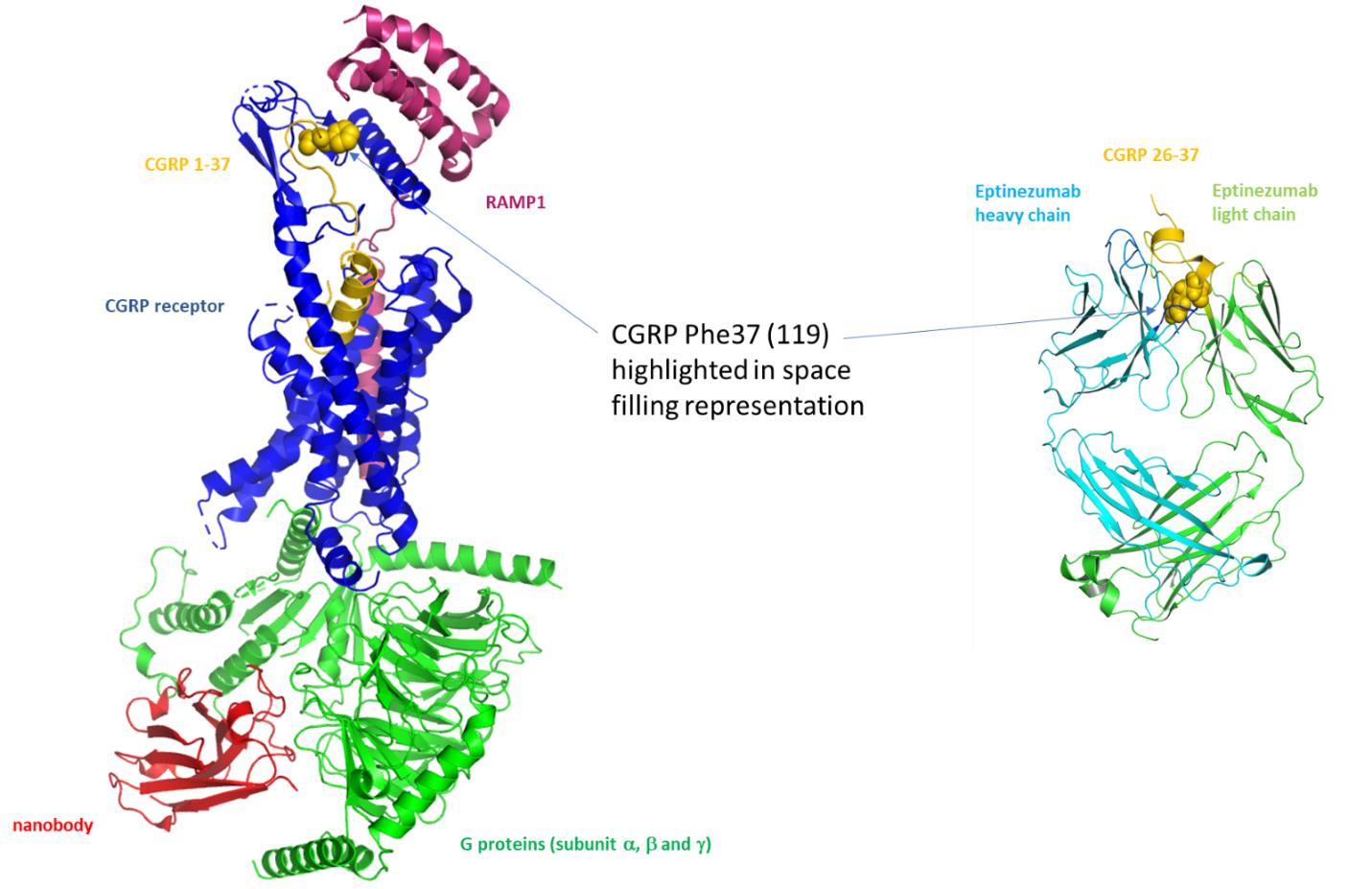
**
